# Unmasking Clever Hans predictors and assessing what machines really learn

**DOI:** 10.1038/s41467-019-08987-4

**Published:** 2019-03-11

**Authors:** Sebastian Lapuschkin, Stephan Wäldchen, Alexander Binder, Grégoire Montavon, Wojciech Samek, Klaus-Robert Müller

**Affiliations:** 1 Department of Video Coding & Analytics, Fraunhofer Heinrich Hertz Institute, Einsteinufer 37, 10587 Berlin, Germany; 2 Department of Electrical Engineering and Computer Science, Technische Universität Berlin, Marchstr. 23, 10587 Berlin, Germany; 3 ISTD Pillar, Singapore University of Technology and Design, 8 Somapah Rd, Singapore, 487372 Singapore; 4 Department of Brain and Cognitive Engineering, Korea University, Anam-dong, Seongbuk-ku Seoul, 136-713 Republic of Korea; 5 Max Planck Institut für Informatik, Campus E1 4, Stuhlsatzenhausweg, 66123 Saarbrücken, Germany

## Abstract

Current learning machines have successfully solved hard application problems, reaching high accuracy and displaying seemingly intelligent behavior. Here we apply recent techniques for explaining decisions of state-of-the-art learning machines and analyze various tasks from computer vision and arcade games. This showcases a spectrum of problem-solving behaviors ranging from naive and short-sighted, to well-informed and strategic. We observe that standard performance evaluation metrics can be oblivious to distinguishing these diverse problem solving behaviors. Furthermore, we propose our semi-automated Spectral Relevance Analysis that provides a practically effective way of characterizing and validating the behavior of nonlinear learning machines. This helps to assess whether a learned model indeed delivers reliably for the problem that it was conceived for. Furthermore, our work intends to add a voice of caution to the ongoing excitement about machine intelligence and pledges to evaluate and judge some of these recent successes in a more nuanced manner.

## Abstract (web summary)

Nonlinear machine learning methods have good predictive ability but the lack of transparency of the algorithms can limit their use. Here the authors investigate how these methods approach learning in order to assess the dependability of their decision making.

## Introduction

Artificial intelligence systems, based on machine learning (ML), are increasingly assisting our daily life. They enable industry and the sciences to convert a never ending stream of data—which per se is not informative—into information that may be helpful and actionable. ML has become a basis of many services and products that we use.

**Fig. 1:**
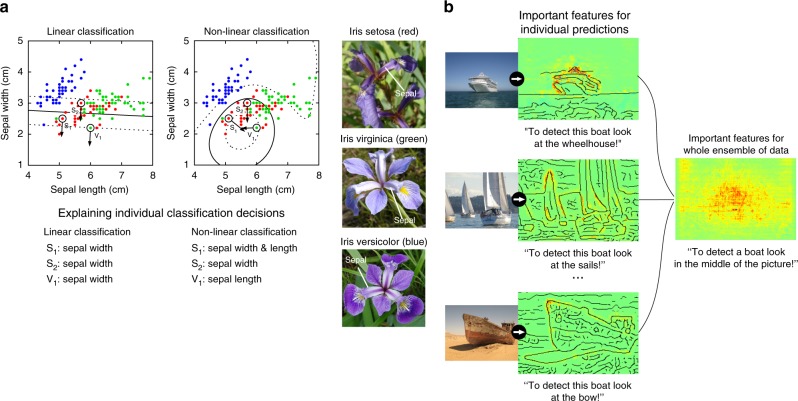
Explanation of a linear and non-linear classifier. **a** In linear models the importance of each feature is the same for every data point. It can be expressed in the weight vector perpendicular to the decision surface where more important features have larger weights. In nonlinear models the important features can be different for every data point. In this example, the classifiers are trained to separate “Iris setosa” (red dots) from “Iris virginica” (green dots) and “Iris versicolor” (blue dots). The linear model for all examples uses the sepal width as discriminative feature, whereas the non-linear classifier uses different combinations of sepal width and sepal length for every data point. **b** Different features can be important (here for a deep neural network) to detect a ship in an image. For some ships, the wheelhouse is a good indicator for class “ship”, for others the sails or the bow is more important. Therefore individual predictions exhibit very different heatmaps (showing the most relevant locations for the predictor). In feature selection, one identifies salient features for the whole ensemble of training data. For ships (in contrast to e.g. airplanes) the most salient region (average of individual heatmaps) is the center of the image

While it is broadly accepted that the nonlinear ML methods being used as predictors to maximize some prediction accuracy, are effectively (with few exceptions, such as shallow decision trees) black boxes; this intransparency regarding explanation and reasoning is preventing a wider usage of nonlinear prediction methods in the sciences (see Fig. 1a why understanding nonlinear learning machines is difficult). Due to this black-box character, a scientist may not be able to extract deep insights about what the nonlinear system has learned, despite the urge to unveil the underlying natural structures. In particular, the conclusion in many scientific fields has so far been to prefer linear models^1–4^ in order to rather gain insight (e.g. regression coefficients and correlations) even if this comes at the expense of predictivity.

Recently, impressive applications of ML in the context of complex games (Atari games^5,6^, Go^7–9^, Texas hold’em poker^10^) have led to speculations about the advent of ML systems embodying true “intelligence”. In this note we would like to argue that for validating and assessing machine behavior, independent of the application domain (sciences, games, etc.), we need to go beyond predictive performance measures, such as the test set error, towards understanding the AI system.

**Fig. 2:**
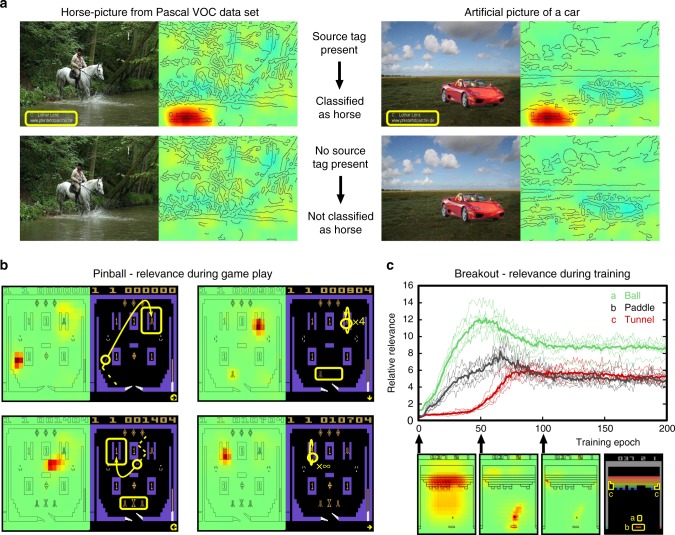
Assessing problem-solving capabilities of learning machines using explanation methods. **a** The Fisher vector classifier trained on the PASCAL VOC 2007 data set focuses on a source tag present in about one-fifth of the horse figures. Removing the tag also removes the ability to classify the picture as a horse. Furthermore, inserting the tag on a car image changes the classification from car to horse. **b** A neural network learned to play the Atari Pinball game. The model moves the pinball into a scoring switch four times to activate a multiplier (indicated as symbols marked in yellow box) and then maneuvers the ball to score infinitely. This is done purely by “nudging the table” and not by using the flippers. In fact, heatmaps show that the flippers are completely ignored by the model throughout the entire game, as they are not needed to control the movement of the ball. **c** Development of the relative relevance of different game objects in Atari Breakout over the training time. Relative relevance is the mean relevance of pixels belonging to the object (ball, paddle, tunnel) divided by the mean relevance of all pixels in the frame. Thin lines: six training runs. Thick line: average over the six runs

When assessing machine behavior, the general task solving ability must be evaluated (e.g. by measuring the classification accuracy, or the total reward of a reinforcement learning system). At the same time it is important to comprehend the decision-making process itself. In other words, transparency of the what and why in a decision of a nonlinear machine becomes very effective for the essential task of judging whether the learned strategy is valid and generalizable or whether the model has based its decision on a spurious correlation in the training data (see Fig. 2a). In psychology the reliance on such spurious correlations is typically referred to as the Clever Hans phenomenon^11^. A model implementing a ‘Clever Hans’-type decision strategy will likely fail to provide correct classification and thereby usefulness once it is deployed in the real world, where spurious or artifactual correlations may not be present.

While feature selection has traditionally explained the model by identifying features relevant for the whole ensemble of training data^12^ or some class prototype^13–16^, it is often necessary, especially for nonlinear models, to focus the explanation on the predictions of individual examples (see Fig. 1b). A recent series of work^13,17–22^ has now begun to explain the predictions of nonlinear ML methods in a wide set of complex real-world problems (e.g. refs. ^23–26^). Individual explanations can take a variety of forms: An ideal (and so far not available) comprehensive explanation would extract the whole causal chain from input to output. In most works, reduced forms of explanation are considered, typically, collection of scores indicating the importance of each input pixel/feature for the prediction (note that computing an explanation does not require to understand neurons individually). These scores can be rendered as visual heatmaps (relevance maps) that can be interpreted by the user.

In the following we make use of this recent body of work, in particular, the layer-wise relevance propagation (LRP) method^18^ (cf. section “Layer-wise relevance propagation”), and discuss qualitatively and quantitatively, for showcase scenarios, the effectiveness of explaining decisions for judging whether learning machines exhibit valid and useful problem solving abilities. Explaining decisions provides an easily interpretable and computationally efficient way of assessing the classification behavior from few examples (cf. Figure 2a). It can be used as a complement or practical alternative to a more comprehensive Turing test^27^ or other theoretical measures of machine intelligence^28–30^. In addition, the present work contributes by further embedding these explanation methods into our framework SpRAy (spectral relevance analysis) that we present in section “Methods”. SpRAy, on the basis of heatmaps, identifies in a semi-automated manner a wide spectrum of learned decision behaviors and thus helps to detect the unexpected or undesirable ones. This allows one to systematically investigate the classifier behavior on whole large-scale datasets—an analysis which would otherwise be practically unfeasible with the human tightly in the loop. Our semi-automated decision anomaly detector thus addresses the last mile of explanation by providing an end-to-end method to evaluate ML models beyond test set accuracy or reward metrics.

## Results

### Identifying valid and invalid problem-solving behaviors

In this section, we will investigate several showcases that demonstrate the effectiveness of explanation methods like LRP and SpRAy for understanding and validating the behavior of a learned model.

First, we provide an example where the learning machine exploits an unexpected spurious correlation in the data to exhibit what humans would refer to as “cheating”. The first learning machine is a model based on Fisher vectors (FV)^31,32^ trained on the PASCAL VOC 2007 image dataset^33^ (see Supplementary Note 5). The model and also its competitor, a pretrained deep neural network (DNN) that we fine-tune on PASCAL VOC, show both excellent state-of-the-art test set accuracy on categories, such as ‘person’, ‘train’, ‘car’, or ‘horse’ of this benchmark (see Supplementary Table 3). Inspecting the basis of the decisions with LRP, however, reveals for certain images substantial divergence, as the heatmaps exhibiting the reasons for the respective classification could not be more different. Clearly, the DNN’s heatmap points at the horse and rider as the most relevant features (see Supplementary Figure 11). In contrast, FV’s heatmap is most focused onto the lower left corner of the image, which contains a source tag. A closer inspection of the data set (of 9963 samples^33^) that typically humans never look through exhaustively, shows that such source tags appear distinctively on horse images; a striking artifact of the dataset that so far had gone unnoticed^34^. Therefore, the FV model has ‘overfitted’ the PASCAL VOC dataset by relying mainly on the easily identifiable source tag, which incidentally correlates with the true features, a clear case of ‘Clever Hans’ behavior. This is confirmed by observing that artificially cutting the source tag from horse images significantly weakens the FV model’s decision while the decision of the DNN stays virtually unchanged (see Supplementary Figure 11). If we take instead a correctly classified image of a Ferrari and then add to it a source tag, we observe that the FV’s prediction swiftly changes from ‘car’ to ‘horse’ (cf. Figure 2a) a clearly invalid decision (see Supplementary Note 5 and Supplementary Figures 12–17 for further examples and analyses).

The second showcase example studies neural network models (see Supplementary Figure 2 for the network architecture) trained to play Atari games, here Pinball. As shown in ref. ^5^, the DNN achieves excellent results beyond human performance. Like for the previous example, we construct LRP heatmaps to visualize the DNN’s decision behavior in terms of pixels of the pinball game. Interestingly, after extensive training, the heatmaps become focused on few pixels representing high-scoring switches and loose track of the flippers. A subsequent inspection of the games in which these particular LRP heatmaps occur, reveals that DNN agent firstly moves the ball into the vicinity of a high-scoring switch without using the flippers at all, then, secondly, “nudges” the virtual pinball table such that the ball infinitely triggers the switch by passing over it back and forth, without causing a tilt of the pinball table (see Fig. 2b and Supplementary Figure 3 for the heatmaps showing this point, and also Supplementary Movie 1). Here, the model has learned to abuse the “nudging” threshold implemented through the tilting mechanism in the Atari Pinball software. From a pure game scoring perspective, it is indeed a rational choice to exploit any game mechanism that is available. In a real pinball game, however, the player would go likely bust since the pinball machinery is programmed to tilt after a few strong movements of the whole physical machine.

The above cases exemplify our point, that even though test set error may be very low (or game scores very high), the reason for it may be due to what humans would consider as cheating rather than valid problem-solving behavior. It may not correspond to true performance when the latter is measured in a real-world environment, or when other criteria (e.g. social norms which penalize such behavior^35^) are incorporated into the evaluation metric. Therefore, explanations computed by LRP have been instrumental in identifying this fine difference.

Let us consider a third example where we can beautifully observe learning of strategic behavior: A DNN playing the Atari game of Breakout^5^ (see Supplementary Table 2 for the investigated network architectures). We analyze the learning progress and inspect the heatmaps of a sequence of DNN models in Fig. 2c. The heatmaps reveal conspicuous structural changes during the learning process. In the first learning phase the DNN focuses on ball control, the handle becomes salient as it learns to target the ball and in the final learning phase the DNN focuses on the corners of the playing field (see Fig. 2c). At this stage, the machine has learned to dig tunnels at the corners (also observed in ref. ^5^)—a very efficient strategy also used by human players. Detailed analyses using the heatmap as a function of a single game and comparison of LRP to sensitivity analysis explanations, can be found in the Supplementary Figures 4–10 and in the Supplementary Movie 2. Here, this objectively measurable advancement clearly indicates the unfolding of strategic behavior.

Overall, while in each scenario, reward maximization, as well as incorporating a certain degree of prior knowledge has done the essential part of inducing complex behavior, our analysis has made explicit that (1) some of these behaviors incorporate strategy, (2) some of these behaviors may be human-like or not human-like, and (3) in some case, the behaviors could even be considered as deficient and not acceptable, when considering how they will perform once deployed. Specifically, the FV-based image classifier is likely to not detect horses on the real-world data; and the Atari Pinball AI might perform well for some time, until the game is updated to prevent excessive nudging.

All insights about the classifier behavior obtained up to this point of this study require the analysis of individual heatmaps by human experts, a laborious and costly process which does not scale well.

### Whole-dataset analysis of classification behavior

**Fig. 3:**
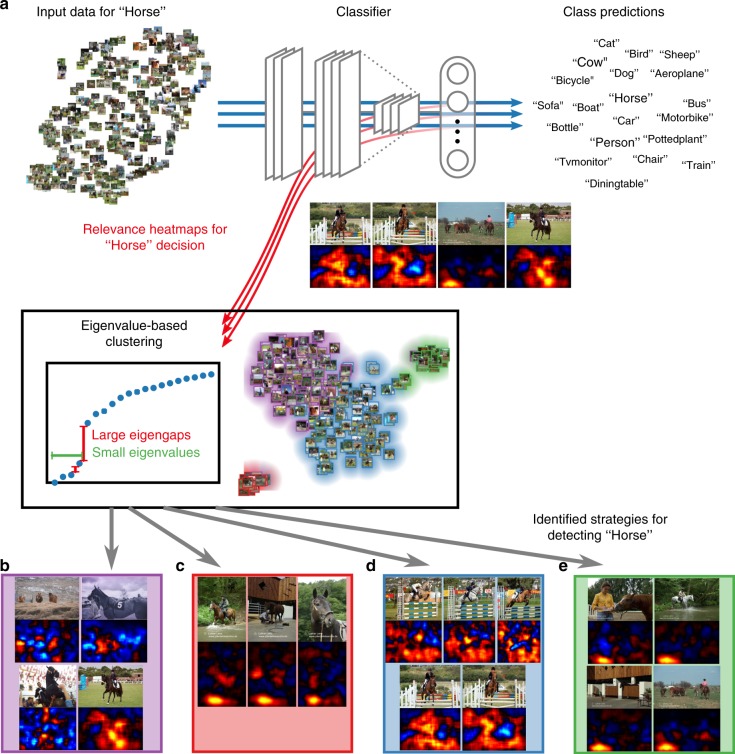
The workflow of spectral relevance analysis. **a** First, relevance maps are computed for data samples and object classes of interest, which requires a forward and a LRP backward pass through the model (here a Fisher vector classifier). Then, an eigenvalue-based spectral cluster analysis is performed to identify different prediction strategies within the analyzed data. Visualizations of the clustered relevance maps and cluster groupings supported by t-SNE inform about the valid or anomalous nature of the prediction strategies. This information can be used to improve the model or the dataset. Four different prediction strategies can be identified for classifying images as “horse”: **b** detect a horse (and rider), **c** detect a source tag in portrait oriented images, **d** detect wooden hurdles and other contextual elements of horseback riding, and **e** detect a source tag in landscape-oriented images

Our next experiment uses SpRAy to comprehend the predicting behavior of the classifier on large datasets in a semi-automated manner. Figure 3a displays the results of the SpRAy analysis when applied to the horse images of the PASCAL VOC dataset (see also Supplementary Figures 19 and 20). Four different strategies can be identified for classifying images as “horse”: (1) detect a horse and rider (Fig. 3b), (2) detect a source tag in portrait-oriented images (Fig. 3c), (3) detect wooden hurdles and other contextual elements of horseback riding (Fig. 3d), and (4) detect a source tag in landscape-oriented images (Fig. 3e). Thus, without any human interaction, SpRAy provides a summary of what strategies the classifier is actually implementing to classify horse images. An overview of the FV and DNN strategies for the other classes and for the Atari Pinball and Breakout game can be found in Supplementary Figures 23–25 and 30–32, respectively.

The SpRAy analysis could furthermore reveal another ‘Clever Hans’-type behavior in our fine-tuned DNN model, which had gone unnoticed in previous manual analysis of the relevance maps. The large eigengaps in the eigenvalue spectrum of the DNN heatmaps for class “aeroplane” indicate that the model uses very distinct strategies for classifying aeroplane images (see Supplementary Figure 23). A t-SNE visualization (Supplementary Figure 25) further highlights this cluster structure. One unexpected strategy we could discover with the help of SpRAy is to identify aeroplane images by looking at the artificial padding pattern at the image borders, which for aeroplane images predominantly consists of uniform and structureless blue background. Note that padding is typically introduced for technical reasons (the DNN model only accepts square-shaped inputs), but unexpectedly (and unwantedly) the padding pattern became part of the model’s strategy to classify aeroplane images. Subsequently we observe that changing the manner in which padding is performed has a strong effect on the output of the DNN classifier (see Supplementary Figures 26–29).

We note that while recent methods (e.g. ref. ^36^) have characterized whole-dataset classification behavior based on decision similarity (e.g. cross-validation-based AP scores or recall), the SpRAy method can pinpoint divergent classifier behavior even when the predictions look the same. The specificity of SpRAy over previous approaches is thus its ability to ground predictions to input features, where classification behavior can be more finely characterized. A comparison of both approaches is given in Supplementary Note 6 and Supplementary Figures 21 and 22.

## Discussion

Although learning machines have become increasingly successful, they often behave very differently from humans^37,38^. Commonly discussed ingredients to make learning machines act more human-like are, e.g. compositionality, causality, learning to learn^39–41^, and also an efficient usage of prior knowledge or invariance structure (see e.g. refs. ^42–44^). Our work adds a dimension that has so far not been a major focus of the machine intelligence discourse, but that is instrumental in verifying the correct behavior of these models, namely explaining the decision making. We showcase the behavior of learning machines for two application fields: computer vision and arcade gaming (Atari), where we explain the strategies embodied by the respective learning machines. We find a surprisingly rich spectrum of behaviors ranging from strategic decision making (Atari Breakout) to ‘Clever Hans’ strategies or undesired behaviors, here, exploiting a dataset artifact (tags in horse images), a game loophole (nudging in Atari Pinball), and a training artifact (image padding). These different behaviors go unnoticed by common evaluation metrics, which puts a question mark to the current broad and sometimes rather unreflected usage of ML in all application domains in industry and in the sciences.

With the SpRAy method we have proposed a tool to systematize the investigation of classifier behavior and identify the broad spectrum of prediction strategies. The SpRAy analysis is scalable and can be applied to large datasets in a semi-automated manner. We have demonstrated that SpRAy easily finds the misuse of the source tag in horse images, moreover and unexpectedly, it has also pointed us at a padding artifact appearing in the final fine-tuning phase of the DNN training. This artifact resisted a manual inspection of heatmaps of all 20 PASCAL VOC classes, and was only later revealed by our SpRAy analysis. This demonstrates the power of an automated, large-scale model analysis. We believe that such analysis is a first step towards confirming important desiderata of AI systems, such as trustworthiness, fairness, and accountability in the future, e.g. in context of regulations concerning models and methods of artificial intelligence, as via the general data protection regulation (GDPR)^45,46^. Our contribution may also add a further perspective that could in the future enrich the ongoing discussion, whether machines are truly “intelligent”.

Finally, in this paper we posit that the ability to explain decisions made by learning machines allows us to judge and gain a deeper understanding of whether or not the machine is embodying a particular strategic decision making. Without this understanding we can merely monitor behavior and apply performance measures without possibility to reason deeper about the underlying learned representation. The insights obtained in this pursuit may be highly useful when striving for better learning machines and insights (e.g. ref. ^47^) when applying ML in the sciences.

## Methods

### Layer-wise relevance propagation

LRP^18^ is a method for explaining the predictions of a broad class of ML models, including state-of-the-art neural networks and kernel machines. It has been extensively applied and validated on numerous applications^23,26,34,48–50^. The LRP method decomposes the output of the nonlinear decision function in terms of the input variables, forming a vector of input features scores that constitutes our ‘explanation’. Denoting **x** = (*x*_1_,…,*x*_*d*_) an input vector and *f*(**x**) the prediction at the output of the network, LRP produces a decomposition **R** = (*R*_1_,…,*R*_*d*_) of that prediction on the input variables satisfying

1 $$\mathop {\sum}\limits_{p = 1}^d R_p = f({\mathbf{x}}).$$

Unlike sensitivity analysis methods^51^, LRP explains the output of the function rather than its local variation^52^ (see Supplementary Note 3 for more information on explanation methods).

The LRP method is based on a backward propagation mechanism applying uniformly to all neurons in the network: Let $a_j = \rho \left( {\mathop {\sum}\nolimits_i a_iw_{ij} + b_j} \right)$ be one such neuron. Let *i* and *j* denote the neuron indices at consecutive layers, and Σ_*i*_, Σ_*j*_ the summation over all neurons in these respective layers. The propagation mechanism of LRP is defined as

2 $$R_i = \mathop {\sum}\limits_j \frac{{z_{ij}}}{{\mathop {\sum}\limits_i z_{ij}}}R_j.$$

where *z*_*ij*_ is the contribution of neuron *i* to the activation *a*_*j*_, and typically depends on the activation *a*_*i*_ and the weight *w*_*ij*_. The propagation rule is applied in a backward pass starting from the neural network output *f*(**x**) until the input variables (e.g. pixels) are reached. Resulting scores can be visualized as a heatmap of same dimensions as the input (see Supplementary Figure 1).

LRP can be embedded in the theoretical framework of deep Taylor decomposition^22^, where some of the propagation rules can be seen as particular instances. Note that LRP rules have also been designed for models other than neural networks, in particular, Bag of Words classifiers, Fisher vector models, and LSTMs (more information can be found in the Supplementary Note 3 and Supplementary Table 1).

### Spectral relevance analysis

Explanation techniques enable inspection of the decision process on a single instance basis. However, screening through a large number of individual explanations can be time consuming. To efficiently investigate classifier behavior on large datasets, we propose a technique: spectral relevance analysis (SpRAy). SpRAy applies spectral clustering^53^ on a dataset of LRP explanations in order to identify typical, as well as atypical decision behaviors of the machine-learning model, and presents them to the user in a concise and interpretable manner.

Technically, SpRAy allows one to detect prediction strategies as identifiable on frequently reoccurring patterns in the heatmaps, e.g., specific image features. The identified features may be truly meaningful representatives of the object class of interest, or they may be co-occurring features learned by the model but not intended to be part of the class, and ultimately of the model’s decision process. Since SpRAy can be efficiently applied to a whole large-scale dataset, it helps to obtain a more complete picture of the classifier behavior and reveal unexpected or ‘Clever Hans’-type decision making.

The SpRAy analysis is depicted in Fig. 3 (see also Supplementary Note 6) and consists of four steps: Step 1: Computation of the relevance maps for the samples of interest. The relevance maps are computed with LRP and contain information about where the classifier is focusing on when classifying the images. Step 2: Downsizing of the relevance maps and make them uniform in shape and size. This reduction of dimensionality accelerates the subsequent analysis, and also makes it statistically more tractable. Step 3: Spectral cluster analysis (SC) on the relevance maps. This step finds structure in the distribution of relevance maps, more specifically it groups classifier behaviors into finitely many clusters (see Supplementary Figure 18 for an example). Step 4: Identification of interesting clusters by eigengap analysis. The eigenvalue spectrum of SC encodes information about the cluster structure of the relevance maps. A strong increase in the difference between two successive eigenvalues (eigengap) indicates well-separated clusters, including atypical classification strategies. The few detected clusters are then presented to the user for inspection. Step 5 (Optional): Visualization by t-stochastic neighborhood embedding (t-SNE). This last step is not part of the analysis strictly speaking, but we use it in the paper in order to visualize how SpRAy works.

Since SpRAy aims to investigate classifier behavior, it is applied to the heatmaps and not to the raw images (see Supplementary Figures 20, 24 and 25 for comparison).

### Code availability

Source code for LRP and sensitivity analysis is available at https://github.com/sebastian-lapuschkin/lrp_toolbox. Source code for Spectral Clustering and t-SNE as used in the SpRAy method is available from the scikit-learn at https://github.com/scikit-learn/scikit-learn. Source code for the Reinforcement-Learning-based Atari Agent is available at https://github.com/spragunr/deep_q_rl. Source code for the Fisher Vector classifier is available at http://www.robots.ox.ac.uk/~vgg/software/enceval_toolkit. Our fine-tuned DNN model can be found at https://github.com/BVLC/caffe/wiki/Model-Zoo#pascal-voc-2012-multilabel-classification-model.

## Supplementary information

Supplementary Information

Description of Additional Supplementary Files

Supplementary Movie 1

Supplementary Movie 2

## Data Availability

The datasets used and analyzed during the current study are available from the following sources. PASCAL VOC 2007: http://host.robots.ox.ac.uk/pascal/VOC/voc2007/#devkit. PASCAL VOC 2012: http://host.robots.ox.ac.uk/pascal/VOC/voc2012/#devkit. Atari emulator: https://github.com/mgbellemare/Arcade-Learning-Environment.
