# Supplementary material for: Unmasking Clever Hans predictors and assessing what machines really learn
Source: Nat Commun. 2019 Mar 11;10:1096. doi: 10.1038/s41467-019-08987-4 (PMC6411769; doi:10.1038/s41467-019-08987-4)
Supplement: Supplementary file 2 — Description of Additional Supplementary Files [file 41467_2019_8987_MOESM2_ESM.docx]

**Description of Supplementary Files**

**File Name:** Supplementary Video 1

**Description:** Video showing the visuals of the Atari Pinball game together with the heatmaps computed by LRP. The agent moves the ball into a scoring switch four times to activate a multiplier and then maneuvers the ball to score infinitely. The heatmap shows that the flippers > Seite 2/2 are completely ignored by the agent throughout the entire game. The agent has learned to control the ball by “nudging” the table.

File Name: Supplementary Video 2

Description: Video showing the visuals of the Atari Breakout game together with the heatmaps computed by Sensitivity Analysis and LRP. Sensitivity Analysis explains a local variation of the agent’s decision function. It produces a result that is noisy and hard to interpret. LRP explains not a variation, but the decision function itself. It distinctly highlights the ball, the tunnel and the paddles, all of which are indeed relevant to the agent’s decision.
